# Urbanisation Drives Microevolution in the Egyptian Fruit Bat (*Rousettus aegyptiacus*)

**DOI:** 10.1111/eva.70243

**Published:** 2026-04-24

**Authors:** Yomiran Nissan, Shani Inbar, Eyal Privman, Yossi Yovel, Orly Razgour

**Affiliations:** ^1^ School of Zoology, George S. Wise Faculty of Life Sciences Tel Aviv University Tel Aviv Israel; ^2^ Department of Evolutionary and Environmental Biology Institute of Evolution, University of Haifa Haifa Israel; ^3^ Sagol School of Neuroscience Tel Aviv University Tel Aviv Israel; ^4^ Biosciences University of Exeter Exeter UK

**Keywords:** ddRAD‐seq, genetic adaptation, genetic homogeneity, genotype‐environment association, landscape genetics, urbanisation

## Abstract

Urbanisation is a pervasive global phenomenon that exerts strong influence on biodiversity and ecosystems. Many species can thrive in urban landscapes by capitalising on generalist traits and environmental resilience; however, this does not safeguard against potential biases exerted by urban environments on population processes. The Egyptian fruit bat ( *Rousettus aegyptiacus* ) is a species with both urban and rural distribution across its range, and some populations show behavioural and physiological differences. Using reduced representation genome sequencing (ddRAD‐seq), we tested for genetic underpinnings of these differences between urban and rural bat populations sampled across Israel. Despite a genetically homogenous landscape presenting no population structure, we show clear isolation by distance and landscape effects on genetic connectivity, where open areas, but not urbanisation, constitute a barrier to movement. Using genotype‐environment association analysis, we identify 59 candidate SNPs spanning 56 genes potentially associated with urbanisation. This suite of genes entails wide‐ranging functions including neurotransmission, metabolism, gene expression regulation, reproductive biology, and retinoic acid and sensory function. Gene Ontology enrichment analysis revealed non‐random functional clustering with exceptional enrichment in GABAergic synapse components (98.6‐fold), monoatomic ion transport (122.2‐fold), and ATP‐dependent chromatin remodelling (68.6‐fold), evidencing coordinated selection across interconnected neural, metabolic, and regulatory systems. A predominance of intronic variants within this candidate SNP suite (51/59) is suggestive that adaptation in response to urbanisation proceeds primarily through changes in gene regulation, rather than protein‐coding modifications. This study shows how a highly mobile species may undergo microevolutionary shifts in response to urban pressures despite ongoing gene flow, elucidating the complex interplay between genetics and the urban environment in a non‐model organism.

## 1 Introduction

Among ecological processes, urbanisation is one of the most important drivers of biodiversity and habitat at a global scale (McKinney 2006; Palomino and Carrascal 2007; Grimm et al. 2008; Shochat et al. 2010). The urban environment involves a number of new challenges such as resource changes, light and noise pollution, and fragmented habitats, among others (Grimm et al. 2008; Alberti et al. 2017). However, some species can well perform in human‐modified habitats (Aronson et al. 2014). Those are mostly generalist species that can tolerate a very wide range of environmental conditions and be abundant in degraded or edge habitats. Since they are not strictly specialised, they can stand rapid changes in their environmental conditions. Generalist species that thrive in urban environments include rats (Ruffino et al. 2011), cockroaches (Adams and Pennings 2022), wild boars (Rosalino et al. 2022), and some bat species (Bateman and Fleming 2012; Ancillotto et al. 2015; Egert‐Berg et al. 2021; Kohyt et al. 2021; Starik and Göttert 2022). The urban environment can provide them with several benefits: reduced predation risk due to the elimination of most natural predators, additional shelters provided by human‐made structures, new foraging opportunities like garbage, planted gardens, or even ecological traps for prey (Bateman and Fleming 2012; Ancillotto et al. 2015; Daniels et al. 2019; Callaghan et al. 2021).

Animals living in cities consistently show different behaviours compared with their rural counterparts. Urban animals are often bolder and less fearful of novel objects (Ana Catarina Miranda et al. 2013). They show enhanced problem‐solving abilities and are more likely to innovate when facing new challenges (Sol et al. 2013; Audet et al. 2016). Their foraging strategies change to exploit human‐provided foods, and many shift their activity patterns to avoid human disturbance (Ditchkoff et al. 2006; Lowry et al. 2013). For example, the differences are striking in Egyptian fruit bats (Egert‐Berg et al. 2021; Harten et al. 2021). These documented changes in behaviour beg a basic question: are these simply flexible responses to the environment or do they reflect genetic adaptation to urban life?

There is a growing number of studies that support the notion that urbanisation can result in genetic adaptation among wild populations. Genetic changes are detectable in species with generation times ranging from 1 to 5 years after urban exposure, often within decades (Hendry et al. 2008; Mueller et al. 2013; Theodorou et al. 2018). Urban white‐footed mice show genetic changes in genes pertaining to metabolism and immune function (Harris et al. 2013). Dark‐eyed junco genes vary between urban and rural populations in loci affecting serotonin signalling and are correlated with reduced stress responses in this species (Atwell et al. 2012). Great tits from cities display selection on genes related to exploratory behaviour (Riyahi et al. 2015). These changes may enable species to better cope in new environments and could contribute to the eventual evolution of distinct urban forms (Johnson and Munshi‐South 2017).

Gene flow between populations has conventionally been considered a constraint to local adaptation, potentially homogenising allele frequencies and acting against selection (Slatkin 1987; Lenormand 2002). However, recent genomic studies indicate a much more nuanced role for gene flow in adaptation (Tigano and Friesen 2016). Gene flow can facilitate adaptation by introducing beneficial genetic variation, enhancing standing genetic variation, and promoting adaptive introgressions across populations (Tigano and Friesen 2016). Populations can maintain adaptive differences under sufficiently strong selection with continued gene flow, with the balance between these depending critically on genetic architecture, including such factors as the effect size of adaptive alleles, physical linkage, and genomic mechanisms reducing recombination (Yeaman and Whitlock 2011; Tigano and Friesen 2016). This balance is particularly interesting in mobile species that can easily move between habitats yet are still under differing selection pressures.

Bats are the only mammals capable of powered flight (Anderson and Ruxton 2020), and some bat species can travel very far distances in a single night (Goldshtein et al. 2020; Toledo et al. 2020). This mobility should facilitate gene flow between populations, which may prevent local adaptation. Yet, many bat species exhibit strong fidelity to their roosts and foraging areas, which may create population structure despite their ability to fly (Kerth et al. 2011). Although many bat species are closely associated with intact natural habitats, some species appear to have adapted to urban living. The Kuhl's pipistrelle ( *Pipistrellus kuhlii* ) seems to benefit from proximity to humans throughout Europe, feeding on insects attracted to streetlights (Ancillotto et al. 2015). A comparative study of genetic connectivity between mice and bats across urban–rural gradients found that bats maintained greater gene flow relative to terrestrial mammals, with flight aiding this in overcoming urban barriers to movement (Richardson et al. 2020). It remains to be seen whether bats are able to adapt to urban environments in the face of such gene flow.

An example of a species that thrives in urban environments across the state of Israel is the Egyptian fruit bat (Korine et al. 1999). This large fruit bat has colonised cities while sustaining rural populations, making it an ideal system in which to study adaptations to urban environments. Recent behavioural studies have demonstrated remarkable differences between urban and rural Egyptian fruit bats (Egert‐Berg et al. 2021; Harten et al. 2021; Weinberg et al. 2025), suggestive of adaptation to city life.

Urban Egyptian fruit bat pups are much bolder and learn much faster than rural pups. Most astonishingly, in cross‐fostering experiments where pups were transferred between urban and rural mothers immediately after birth, urban pups raised by rural mothers stayed bold while rural pups raised by urban mothers exhibited a resemblance to their foster mothers in relation to their level of risk‐taking compared to their biological mothers. The rural pups fostered by urban mothers were found to be bolder than those that were fostered by rural mothers, who in turn had lower levels of risk‐taking (Harten et al. 2021). These results point to a post‐birth maternal effect, potentially mediated through hormonal transfer, though they do not exclude additional genetic or epigenetic components. The dietary diversity of urban bat foragers was significantly higher than that of their rural counterparts, with higher Shannon and Simpson diversity indices due to the consumption of ornamental trees including date palms, Ficus spp. and exotic fruits. GPS tracking revealed that rural bats commute up to 25 km to forage in cities while discovering urban food sources independently rather than following other bats, indicating individual variation in exploratory behaviour with potential genetic background (Egert‐Berg et al. 2021).

Physiological measures indicated another important difference. The cortisol in the milk of urban mothers is much higher than among rural mothers (Harten et al. 2021). Cortisol is a hormone associated with stress and influences metabolism, immunity, and behaviour (Sapolsky et al. 2000). The transfer of cortisol may thus be one way in which mothers prepare their pups for city life, since maternal hormone transfer has been shown to influence the development of offspring behavioural phenotypes (Harten et al. 2021). Most recently, urban female fruit bats were found to give birth roughly 2.5 weeks earlier in spring compared to rural females (presumably due to higher ambient temperatures, abundant year‐round food resources from ornamental trees, and more protected roost environments). This earlier parturition timing may enable the completion of a second annual reproductive cycle late in summer by urban females, for which only 70%–80% of females often achieve biannual reproduction. Significantly, despite this temporal shift, urban bats achieve comparable reproductive success compared to rural populations, indicating a true adaptation rather than a costly trade‐off (Weinberg et al. 2025).

Egyptian fruit bats have exceptional navigational capabilities that may be of particular value in cities. They are able to find their way back to the roost from distances of over 80 km using a cognitive map based on visual landmarks (Tsoar et al. 2011). This cognitive map is developed by young bats as they start to go on exploratory flights, learning the surrounding landscape progressively beyond the roost (Harten et al. 2020). They integrate all the visual information with echolocation and probably magnetic cues when navigating (Toledo et al. 2020). Such advanced spatial cognition helps them navigate through the complex urban environments with buildings, roads, and food resources that can be patchy.

These differences in behaviour and physiology, along with continued gene flow between urban and rural Egyptian fruit bat populations, create an ideal system to study microevolution in response to urbanisation. The observed behavioural flexibility of urban bats, their changed stress physiology, and dietary adaptations suggest that various biological systems have responded to urban selection pressures. Understanding whether these differences have a genetic basis is crucial for predicting how species will respond to continued urban expansion. A previous genetic study using 18 microsatellite markers and circuit theory modelling found no population structure between urban and rural Egyptian fruit bats in Israel and concluded that habitat use did not significantly predict gene flow beyond geographic distance (Centeno‐Cuadros et al. 2017). However, neutral markers may not detect adaptive genetic variation at loci under selection (Funk et al. 2012), and modern genomic approaches with thousands of variants can reveal signatures of selection even when neutral population structure is absent (Rellstab et al. 2015). Moreover, that landscape framework did not incorporate variables such as artificial light intensity, continuous tree cover, or orchard proximity that may more directly influence movement in this species.

The goal of this study is to investigate the impact of urbanisation on highly mobile mammals and to find a potential genetic basis for the documented behavioural and physiological differences between urban and rural Egyptian fruit bats. Here, we investigate the genetic composition of urban versus rural populations using double digest restriction site‐associated DNA sequencing (ddRAD‐seq). This method enables us to genotype many individuals at thousands of loci across the genome, thus providing power to detect both population structure and signatures of selection (Peterson et al. 2012; Andrews et al. 2016).

We hypothesise that even in the face of high mobility and gene flow among populations, urbanisation imposes selective pressures contributing to genetic differentiation at specific loci. We predict that: (1) neutral genetic structure will exhibit little differentiation between urban and rural populations due to gene flow, consistent with results of prior microsatellite studies (Centeno‐Cuadros et al. 2017); (2) landscape features will impact genetic connectivity, with open areas potentially reducing gene flow; and (3) based on observed behavioural and physiological differences, bats in urban environments will exhibit adaptive variation in genes related to neurotransmission, stress response, and metabolism. Using thousands of genetic markers and associating them with environmental variables, this study will identify the genetic architecture underlying adaptation to urban life in a highly mobile mammal.

## 2 Methods

### 2.1 Study Sites and Sampling

Israel has undergone extensive urban expansion over the past decades. The human population increased from approximately 800,000 in 1948 to more than 9 million currently. This rapid urbanisation has transformed more than 7% of the landscape into built environment by 2009 (Levin et al. 2009), a figure that has likely increased substantially since, causing declines in many species while benefiting others, including Egyptian fruit bats (Mendelssohn and Yom‐Tov 1999). Urban fruit bats exploit extensive plantations of date palms and Ficus trees planted for shade in cities, with more than 100,000 date palms and 50,000 Ficus trees in the Greater Tel Aviv area providing year‐round fruit resources (Weinberg et al. 2023).

We studied Egyptian fruit bats from two main regions in Israel (Figure S1): (1) an urban area in the Greater Tel Aviv (Gush‐Dan) metropolitan (Latitude 32.1, Longitude 34.8) and (2) a rural area around Beit‐Govrin National Park (31.6, 34.9). These sites have been sampled regularly over the past few years to study behavioural differences between the populations (Egert‐Berg et al. 2021). The Gush‐Dan area is the largest metropolitan area in Israel. Here, we define the area as encompassing the 15 km radius around central Tel‐Aviv, where more than 2.5 million people live.

During 2018 we captured bats in 10 roosts with permission from the National Park Authority (number 2018–41995), five roosts in the urban area, and five in the rural area, using butterfly nets or mist nests, and collected 3 mm wing biopsies from ~20 individuals per roost, including both males and females (Table 1). We examined the sex of bats and their reproductive condition and measured their weight and forearm length. Morphometric data were not analysed further in the present study. Once data collection was completed, bats were released back into the wild.

**TABLE 1:** Location of the sampling sites and number of female and male Egyptian fruit bats sampled from each roost.

Area	Colony name	Latitude	Longitude	Urban coverage (%)	Number of females	Number of males
Urban	Herzliya cave	32.168	34.814	64.5	13	6
	Halfred Bridge	32.093	34.801	89.4	16	3
	Dan center, Ramat‐Gan	32.092	34.822	85.8	16	3
	Soncino, Tel‐Aviv	32.066	34.787	87.6	13	6
	Jaffa soap factory	32.054	34.754	90.9	9	10
	Total				67	28
Rural	Twins cave	31.726	35.02	13.1	9	10
	Sgafim cave	31.682	34.909	6.4	13	6
	Luzit cave	31.675	34.885	2.9	9	10
	Beit Guvrin cave	31.6	34.902	2.9	6	12
	Semech cave	31.555	34.812	13.1	11	8
	Total				48	46

### 2.2 DNA Extraction, Library Building, Sequencing and SNP Genotyping

We extracted genomic DNA from wing biopsies using DNeasy Blood & Tissue Kit (QIAGEN Ltd., Germany) with one modification to the suggested protocol, in the elution stage, we used 35 ul of elution buffer in two repetitions to increase yield. After extraction, each sample was tested for DNA concentration using Thermo Scientific NanoDrop 2000. The 19 highly concentrated samples from each colony were used to generate ddRADseq libraries following the (Brelsford et al. 2016) protocol. We use the script from Herrera et al. (2015) on the published Egyptian fruit bat genome from (Pavlovich et al. 2018) and other known whole bat genomes from Fang et al. (2015) to identify the most suitable restriction enzymes for Egyptian fruit bat. Our analysis determined that the 6‐base cutter PstI and the 4‐base cutter MseI (New England Biolabs Ltd., UK) would target the appropriate number of loci for genotyping. To achieve the desired sequencing depth of 10–20×, we calculated the required number of sequencing lanes and performed sequencing using two lanes of Illumina HiSeq X.

Libraries were sent for sequencing using two lanes of Illumina HiSeq X (paired‐end, 2 × 150 bp) at Admera Health Ltd., New‐Jerzy, USA. Illumina raw sequencing data were demultiplexed using the STACKS v2.53 process_radtag script (Catchen et al. 2013) which was also used to extract the different individuals based on their indices and barcodes, and then filtered and trimmed for quality using. BWA‐MEM v0.7.17 (Li and Durbin 2010), was used to aligned the reads to the reference genome ‘mRouAeg1.p’ assembly (Jebb et al. 2020) and SAMTOOLS v1.10 (Li et al. 2009) was used to convert and sort the output files. STACKS v2.53 ‘gstacks’ and ‘populations’ scripts were used to call variants.

To limit false SNP identification and increase the precision of the subsequent analyses, VCFtools v0.1.13 (Danecek et al. 2011) was used to discard SNPs with a minor allele frequency (MAF) < 3%. In addition, we excluded genotypes with a depth under 5, loci with more than 20% missing data, and a mean depth over 50. The last criterion is aimed to exclude repetitive sequences. We used PLINK v1.9 (Purcell et al. 2007) to identify linkage disequilibrium (LD) among the SNPs. We performed aggressive LD‐pruning with a sliding window of 25 SNPs and a step size of 10 SNPs, and removed one SNP from each pair in each window above the LD threshold of *r* ^2^ = 0.9.

### 2.3 Identifying Population Structure

To determine the genetic population structure, we used the snmf function in the R package LEA v3.2.0 (Frichot and François 2015), which performs a Bayesian clustering algorithm similar to STRUCTURE. The number of population groups was set at *K* = 1–5, using 1000 repetitions and 1000,000 iterations for each *K* with the entropy function. Additionally, we performed a principal component analysis (PCA) using the LEA package in R (Frichot and François 2015) on 20,205 LD‐pruned SNPs. We calculated pairwise values of genetic differentiation (*F*_ST_, in accordance with Weir and Cockerham 1984) between the 10 colonies using VCFtools v0.1.13. The output FST values were linearised (*F*_ST_/(1 − *F*_ST_)). The distances between colonies were calculated in QGIS (Dawson et al. 2025). To test for isolation by distance, we performed Multiple Regression on distance Matrices (MRM) analysis instead of a simple Mantel test, as MRM provides a more robust framework for analysing distance matrices through regression rather than correlation alone. The MRM analysis was implemented using the MRM() function from the ecodist R package (Goslee and Urban 2007), with 9999 permutations for significance testing. Additionally, we compared genetic distances within urban colonies (*n* = 10 pairwise comparisons), within rural colonies (*n* = 10), and between urban and rural colonies (*n* = 25) using two‐sample *t*‐tests. To assess levels of heterozygosity and inbreeding, we utilised the het and ibc functions in PLINK v1.9 (Purcell et al. 2007) to calculate observed and expected heterozygosity and the inbreeding coefficient (*F*) for each individual in the dataset.

### 2.4 Landscape Genetics Analysis

We used the landscape genetic approach to identify barriers to genetic connectivity between Egyptian fruit bat colonies in urban and rural areas. We generate resistance surfaces in ArcGIS v10.8 (ESRI) based on five different hypotheses. Resistance costs were assigned based on expert opinion and ranged from one, no resistance to movement, to 100, strong barrier to movement. The resolution of the analysis ranged between 25 and 458 m, depending on the resolution of the original map used to generate the resistance surface (Table S2). We used Circuitscape v4.0.5 (McRae 2006) to calculate resistance distance matrices between the bat colonies based on the cumulative cost of movement due to landscape resistance.

Our five hypotheses compared the effect of four landscape elements thought to potentially affect genetic connectivity. As this study compares urban and rural colonies, the first variable included in our analysis was the extent of artificial lights at night, as an indicator of urbanisation. We hypothesised that for bats from rural colonies, the cost of crossing the landscape matrix will increase with increasing extent of artificial lights. Therefore we assigned resistance costs that increased linearly with light intensity, from lowest costs (resistance costs = 1) for unlit areas. Second, we included the effect of distance to orchards. As these fruit bats forage on fruits from orchards (Hadjisterkotis 2006; Luĉan et al. 2016), we hypothesised that the bats were more likely to travel across areas near orchards, and therefore we assigned resistance cost of 1 (no resistance) to areas with orchards and increasing resistance costs as distance from orchards increased (up to 100 maximum resistance). Third, we looked at the effect of tree cover more generally. As these bats use trees for foraging and navigation (Tsoar et al. 2011; Harten et al. 2020; Toledo et al. 2020), we hypothesised that they are more likely to cross areas with higher percentage of tree cover. Hence, we assigned resistance costs relative to the percent of tree cover with highest resistance costs (100) for areas with no tree cover. Fourth, we looked at the effect of anthropogenic land use change by generating three different scenarios that varied in the costs assigned to crossing urban areas, from high (50; land cover 1) to low costs (10; land cover 3). Orchards and forests were consistently assigned low resistance costs (1–10), while grassland, arable and bare ground were assigned highest resistance costs (80–100). Fifth, we examine the combined effect of artificial lighting and the distance to orchards. Finally, we included the effect of isolation by distance, to test if genetic connectivity is not affected by landscape barriers only by geographic distance between colonies (Table S2 for allocation of landscape resistance costs and map sources).

Landscape resistance distance matrices were related to levels of genetic differentiation (linearised *F*_ST_) between colonies. We first selected the best combination of resistance costs for the land cover hypothesis based on the strength of correlation with the genetic distance between colonies, using the multiple regression on distance matrices function in the R package ecodist (Table S3) (Goslee and Urban 2007). We then used Maximum Likelihood Population Effect models (Van Strien et al. 2012) implemented through the R packages lme4 (Bates et al. 2015) and usdm (Naimi et al. 2014) to compare our different hypotheses based on AICc and BIC evidence weights scores. We estimated the 95% Confidence Intervals for the best supported models to assess whether they overlap zero.

### 2.5 Genotype‐Environmental Associations and Gene Ontology

We carried out genotype‐environment association (GEA) analysis to identify loci potentially under selection from environmental conditions associated with urbanisation. Environmental variables included percent urban cover (extracted from HaMaarag, Israel National Ecosystem Assessment Program, https://www.hamaarag.org.il/) calculated within a 5 km radius around each colony at 1 km resolution.

Analysis was performed using redundancy analysis (RDA) implemented in the vegan 2.6–4 R package (Oksanen et al. 2022). Prior to RDA, missing genotypes were imputed within each cluster by replacing missing values with the rounded mean genotype across individuals within that cluster. RDA was performed on scaled genetic data with urban coverage as the constraining variable. Statistical significance was assessed using permutation tests (999 permutations) for the full model (anova.cca) and individual axes.

SNPs putatively under selection were identified as those with loading scores exceeding ±3 standard deviations from the mean loading on each RDA axis. For each of these SNPs, we calculated Pearson correlation coefficients and associated *p*‐values with urban coverage. Only SNPs showing significant correlations (*p* < 0.05) with urban coverage were retained for further analysis.

SNP annotation was performed using the Egyptian fruit bat reference genome assembly mRouAeg1.p (Jebb et al. 2020) (GCF_014176215.1) and its associated GTF annotation file. Using custom MATLAB scripts, we identified whether each putative urban‐associated SNP was located within genes (introns or exons) or intergenic regions. Genes with “LOC” (unrecognised) prefixes were excluded from analysis. Gene functions were determined using GeneCards (Stelzer et al. 2016), UniProt (Ahmad et al. 2025), KEGG (Kanehisa and Goto 2000), Gene Ontology knowledgebase (Aleksander et al. 2023) and NCBI database (Sayers et al. 2025).

For network analysis, genes showing associations with urbanisation were mapped to human orthologs using the STRING database v12.0 (Szklarczyk et al. 2023) with *Homo sapiens* (taxon 9606) protein interaction data. We used a confidence score threshold of 400 (medium confidence) to filter interactions. Protein–protein interaction networks were constructed and analysed using the igraph (Antonov et al. 2023) package in R. Network topology metrics were calculated including degree centrality, betweenness centrality, closeness centrality, and eigenvector centrality. Hub genes were identified as nodes with degree exceeding the mean plus one standard deviation.

Gene Ontology (GO) enrichment analysis was performed using hypergeometric tests with the Gene Ontology database (Aleksander et al. 2023) of *Homo sapiens* (taxon 9606). For each GO term, we calculated the proportion of genes with that term in our candidate set versus the background frequency across all annotated genes. *p*‐values were corrected for multiple testing using the Benjamini‐Hochberg false discovery rate (FDR) method with a significance threshold of 0.05.

## 3 Results

We performed deep sequencing on 188 individuals (one individual failed during library preparation) and generated nearly 1.56 billion reads in total. After demultiplexing and quality control, we retained approximately 1.13 billion reads with an average of 5,949,566 ± 1,330,048 reads per individual (mean ± SD). We identified 390,582 loci across the genome and retained 20,205 bi‐allelic SNPs from 185 individuals after applying quality control criteria.

### 3.1 Identifying the Population Structure Across Urban and Rural Populations

**FIGURE 1:**
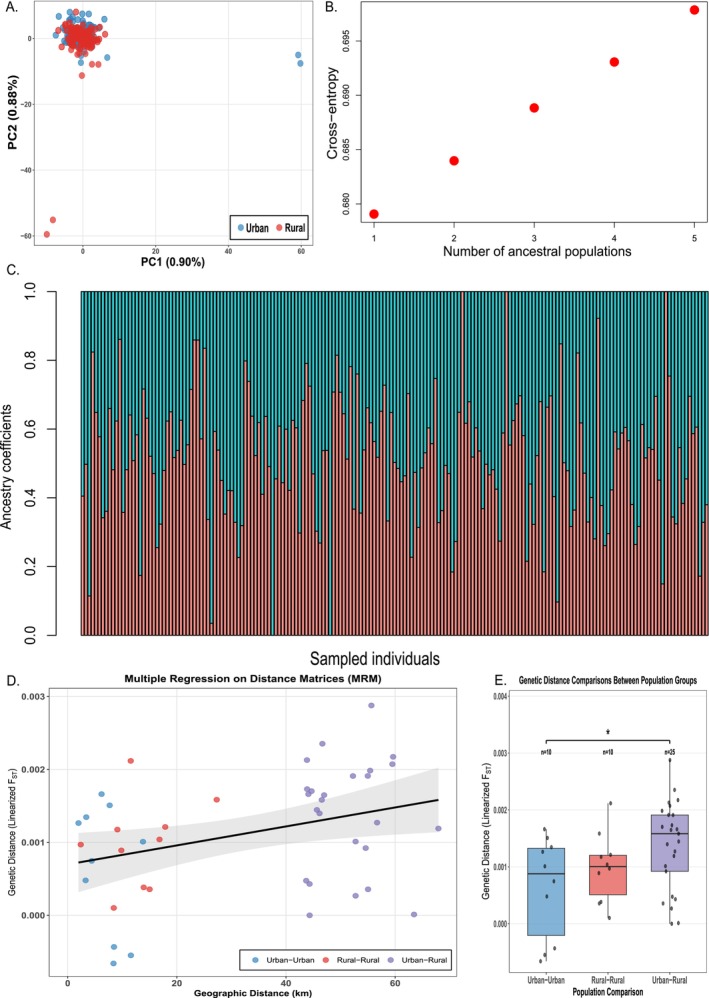
Genetic structure between 185 individuals from urban and rural areas in Israel. (A) Principal component analysis of 185 individuals from 10 colonies showing no distinct clustering between urban (blue) and rural (red) populations. PC1 and PC2 explain 0.90% and 0.88% of the variance, respectively. (B) Cross‐entropy values from SNMF analysis supporting *K* = 1 as the optimal number of genetic clusters. (C) Admixture barplot at *K* = 2 showing lack of population subdivision between urban and rural colonies (shown for visualisation despite *K* = 1 being optimal). (D) Multiple Regression on distance Matrices (MRM) analysis demonstrating isolation by distance. Points are coloured by comparison type: Urban–urban (blue, *n* = 10), rural–rural (red, *n* = 10), and urban–rural (purple, *n* = 25). Black line shows regression fit with 95% confidence interval (grey shading). (E) Comparison of linearised genetic distances (*F*_ST_/(1 − *F*_ST_)) among population groups. Box plots show median (line), interquartile range (box), and range (whiskers), with individual data points overlaid. Asterisk indicates significant difference (*p* = 0.035, two‐sample *t*‐test). Sample sizes indicate number of pairwise comparisons.

Principal component analysis (PCA) revealed no clear genetic clustering between urban and rural populations (Figure 1A). The first and second principal components explained 0.90% and 0.88% of the variance, respectively. Four individuals (two urban, two rural) appeared somewhat separated from the main cluster. These individuals did not show elevated rates of missing genotypes (range: 1.9%–10.0%) relative to the dataset mean (8.1%), ruling out technical artefacts as an explanation. Tracy‐Widom tests indicated that the first four principal components were statistically significant (*p* < 0.001 for PC1‐PC3, *p* = 1.77 × 10^−4^ for PC4), though together they explained only 3.4% of the total variance. This separation did not correspond to any clear geographic or environmental pattern. Admixture analysis supported a single genetic cluster (*K* = 1) as optimal based on cross‐entropy criterion (Figure 1B), with no evidence of population subdivision between urban and rural colonies when visualised at *K* = 2 (Figure 1C).

*F*_ST_ values were uniformly low across all population comparisons, ranging from 0.00003 to 0.0041, with an overall mean of 0.002 ± 0.001, indicating high gene flow among colonies. The average *F*_ST_ for urban–urban comparisons was 0.0016 ± 0.001, for rural–rural comparison was 0.0019 ± 0.001, and for urban–rural comparison was 0.0024 ± 0.001. Observed heterozygosity exceeded expected values in both urban (Ho/He = 1.0167 ± 0.0472) and rural (Ho/He = 1.0092 ± 0.0305) colonies, with corresponding negative inbreeding coefficients (Urban: *F* = −0.0095 ± 0.0319; Rural: *F* = −0.0068 ± 0.0239), suggesting random mating within populations (Table S1). In addition, no genetic differentiation observed between males and females (weighted *F*_ST_ = 9.19 × 10^−5^, based on 182 individuals with known sex), confirming that sex‐specific processes do not confound the urban–rural comparisons.

### 3.2 Isolation by Distance

Multiple Regression on distance Matrices (MRM) analysis revealed significant isolation by distance (Figure 1D). Geographic distance explained 12.1% of the variation in genetic distance (*R* ^2^ = 0.121, *F*_1,43_ = 5.92, *p* = 0.010), with a moderate positive correlation between genetic and geographic distances (*r* = 0.348). The regression indicated that linearised genetic distance increased by 1.30 × 10^−5^ per kilometer of geographic separation (95% CI: 2.23 × 10^−6^ to 2.38 × 10^−5^). Comparison of genetic distances among population groups (Figure 1E) showed that urban–rural comparisons had significantly higher genetic differentiation (mean linearised *F*_ST_ = 0.0014 ± 0.0008) than urban–urban comparisons (mean = 0.0006 ± 0.0009; t(15) = −2.32, *p* = 0.035). Rural–rural comparisons (mean = 0.0010 ± 0.0006) did not differ significantly from either urban–urban (t(16) = −1.02, *p* = 0.32) or urban–rural comparisons (t(21) = −1.62, *p* = 0.12). These patterns correspond to the greater geographic distances between urban and rural colonies (mean = 51.3 km) compared to within‐region comparisons (urban–urban: 6.9 km; rural–rural: 13.2 km; Table 2).

**TABLE 2:** Summary of genetic and geographic distances among population comparison groups.

Population comparison	Mean genetic distance (linearised *F*_ST_)	SD	Mean geographic distance (km)	SD	Statistical comparison
Urban vs. Urban	0.000638	0.0009	6.9	3.8	Reference group
Rural vs. Rural	0.000983	0.0006	13.2	6.7	*p* = 0.32 (ns)
Urban vs. Rural	0.001381	0.0008	51.3	6.9	*p* = 0.035*

*Note:* Mean linearised genetic distance (*F*_ST_/(1 − *F*_ST_)) and geographic distance (km) with standard deviations for pairwise comparisons within and between urban and rural colonies. Statistical comparisons show results of two‐sample *t*‐tests against the urban–urban reference group.

Abbreviation: ns, not significant.

* *p* < 0.05.

### 3.3 Landscape Genetics Analysis

**FIGURE 2:**
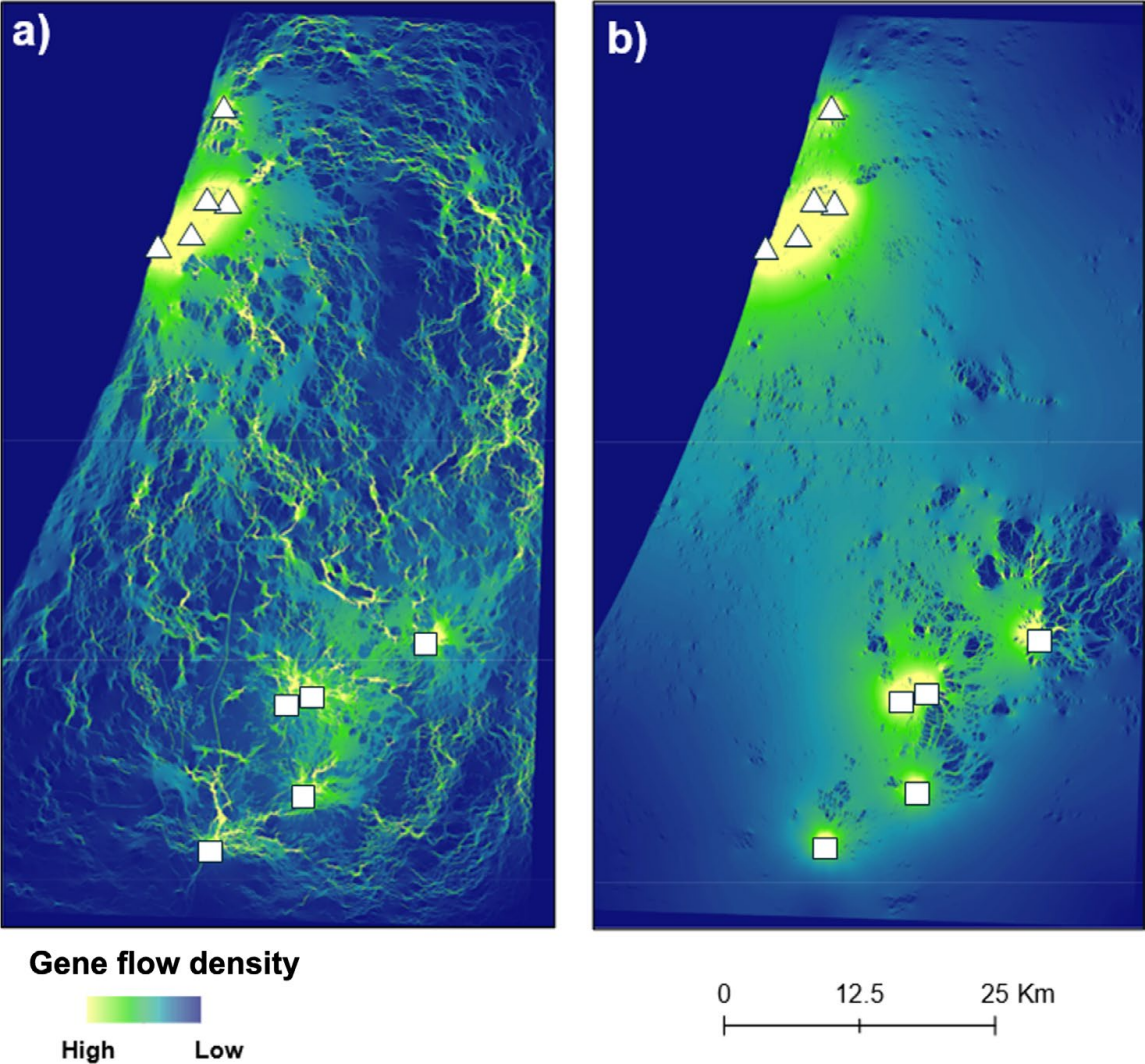
Results of the landscape genetics analysis showing predicted gene flow density potential between Egyptian fruit bat colonies based on the effect of landscape resistance due to (a) land cover, and (b) percent tree cover. Triangles are urban colonies in the Gush‐Dan area, and squares are rural colonies in the Beit‐Guverin national park area. Yellow and green indicate high gene flow potential, while blue colours indicate low gene flow potential.

Of the three land cover variables, the variable assigning lower resistance costs to urban areas (land cover 3) was selected to be included in the analysis because it explained the largest proportion of the variation (*R* ^2^ = 0.177, *p* = 0.015; Table S3). This landscape variable was identified as the most strongly affecting genetic connectivity between Egyptian fruit bat colonies in the landscape genetics analysis (*R* ^2^ = 0.177, AICcmin = 0.332). Based on this variable, orchard, forest, and urban land cover facilitated gene flow, while bare areas, arable land and grasslands were barriers to genetic connectivity. The second best supported hypothesis was percent tree cover (*R* ^2^ = 0.090, AICcmin = 0.316), according to which genetic connectivity increased with increasing tree cover, measured based on canopy closure for all vegetation taller than 5 m. The confidence intervals of both models did not overlap zero, supporting their effect on gene flow. Both models were better supported than the null hypothesis of isolation by distance (Table 3). Gene flow density maps based on the impacts of land cover and percent tree cover show the highest levels of genetic connectivity within urban or rural areas, but also strong connectivity between these areas (Figure 2).

**TABLE 3:** Results of the maximum likelihood population effect models.

Hypotheses	*R* ^2^	AICcmin	BICew	2.5% CI	97.5% CI
H1: Light	0.014	0.136	0.137		
H2: Distance to orchards	0.001	0.002	0.002		
H3: Percent tree cover	0.090	**0.316**	**0.318**	0.000007	0.000025
H4: Land cover 3 (low costs of crossing urban areas)	0.177	**0.332**	**0.334**	0.00003	0.0001
H5: Orchard + Light	0.015	0.034	0.089		
Isolation by Distance	0.122	0.179	0.180		

*Note:* Comparing the five landscape resistance hypotheses and isolation by distance to levels of genetic connectivity (linearised *F*_ST_) between Egyptian fruit bat colonies.

Abbreviations: AICcmin, Akaike Information Criterion corrected for small sample sizes weights; BICew, Bayesian Information Criterion weights.

### 3.4 Genotype‐Environment Association Analysis, Gene Networks, Gene Ontology, and Functional Annotation

Genotype‐environment association analysis using redundancy analysis (RDA) identified SNPs showing allele frequency differentiation associated with urban coverage (Figure S2). Outlier SNPs were detected using a threshold of ±3 standard deviations from the mean loading scores on RDA axes. After filtering for significant associations with urban coverage (*p* < 0.05), 59 SNPs located within or near genes were retained for functional analysis (Table 4; Table S4). These 59 SNPs corresponded to 56 unique genes, as some genes contained multiple SNPs. The strength of association between SNP genotypes and urban coverage ranged from |*r*| = 0.146 to 0.392 (mean |*r*| = 0.276), with *p*‐values ranging from 3.43 × 10^−8^ to 4.78 × 10^−2^ (median *p* = 8.99 × 10^−5^), indicating strong signatures of differential selection between urban and rural environments.

**TABLE 4:** Summary of SNPs that were found to be linked to urbanisation and light intensity.

Gene	Association strength (|*r*|)	*p*	SNP location	Primary function
Gene Expression Regulation				
CHD6	0.343	1.72 × 10^−6^	Intron	Chromatin remodeling
GTF2E1	0.283	9.33 × 10^−5^	Intron	Transcription initiation
RERE	0.148	0.0448	Intron	Transcriptional repression
SLTM	0.146	0.0478	Intron	Transcription modulation
C1D	0.207	0.00473	—	Nuclear matrix organisation
Neurotransmitter function				
GRID1	0.324	6.63 × 10^−6^	Intron	Glutamate receptor activity
SYNGR1	0.343	1.75 × 10^−6^	Intron	Synaptic vesicle function
DGLUCY	0.263	0.000291	Intron	Glutamate metabolism
Hormone/reproductive signaling				
STRA8	0.289	6.51 × 10^−5^	Intron	Retinoic acid response
RAMP1	0.158	0.0313	Intron	Receptor signaling
THEMIS	0.289	6.60 × 10^−5^	Intron	Immune cell development
CLTCL1	0.289	6.68 × 10^−5^	Exon	Clathrin‐mediated transport
CNGB3	0.287	2.75 × 10^−5^	Intron	Cyclic nucleotide signaling
PLCG1	0.287	7.49 × 10^−5^		Phospholipase activity
Metabolism				
ATP8B1	0.262	0.000304	Intron	Lipid transport
PLPPR3	0.268	0.000224	Intron	Lipid metabolism
EPN3	0.389	4.62 × 10^−8^	Exon	Membrane metabolism
B3GAT2	0.375	1.39 × 10^−7^	Intron	Carbohydrate metabolism
CAMK1D	0.323	7.49 × 10^−6^	Intron	Protein kinase activity
Light/circadian				
CLTCL1	0.289	6.68 × 10^−5^	Exon	Light chain binding
CNGB3	0.287	2.75 × 10^−5^	Intron	Photoreceptor function

The genes showing the strongest associations with urbanisation included CFAP58 (|*r*| = 0.392, *p* = 3.43 × 10^−8^), EPN3 (|*r*| = 0.389, *p* = 4.62 × 10^−8^), CACNA1G (|*r*| = 0.383, *p* = 7.13 × 10^−8^), B3GAT2 (|*r*| = 0.375, *p* = 1.39 × 10^−7^), GARNL3 (|*r*| = 0.372, *p* = 1.87 × 10^−7^), CHD6 (|*r*| = 0.343, *p* = 1.72 × 10^−6^), SPATS2L (|*r*| = 0.343, *p* = 1.70 × 10^−6^), and SYNGR1 (|*r*| = 0.343, *p* = 1.75 × 10^−6^). These selection signatures are among the strongest detected in the dataset, with *p*‐values indicating highly significant allele frequency differentiation between populations.

Among the candidate genes, we identified several with clear functional relevance to urbanisation processes. Gene expression regulation genes included CHD6 (chromatin remodelling), GTF2E1 (transcription factor TFIIE), RERE (transcriptional corepressor), SLTM (transcription modulator), and C1D (transcription factor). Neurotransmitter‐related genes included GRID1 (glutamate receptor), DGLUCY (glutamate metabolism), and SYNGR1 (synaptic vesicle protein). Hormone signalling genes included RAMP1 (receptor activity modifying protein), STRA8 (retinoic acid signalling in reproduction), THEMIS (immune/hormone signalling), CLTCL1 (clathrin‐mediated processes), CNGB3 (cyclic nucleotide‐gated channel), and PLCG1 (phospholipase C gamma).

Systematic functional classification revealed 56 candidate genes distributed across multiple urbanisation‐relevant categories based on Gene Ontology annotations. The analysis identified genes with functions related to cellular processes including gene regulation (14 genes), neurotransmitter systems (13 genes), hormone signalling (11 genes), metabolism (3 genes), development (3 genes), and stress response (2 genes). Notably, neurotransmitter genes were strongly represented among genes showing differentiation (13 of 56 genes, 23%), suggesting selection on neural function in response to urban environments.

**FIGURE 3:**
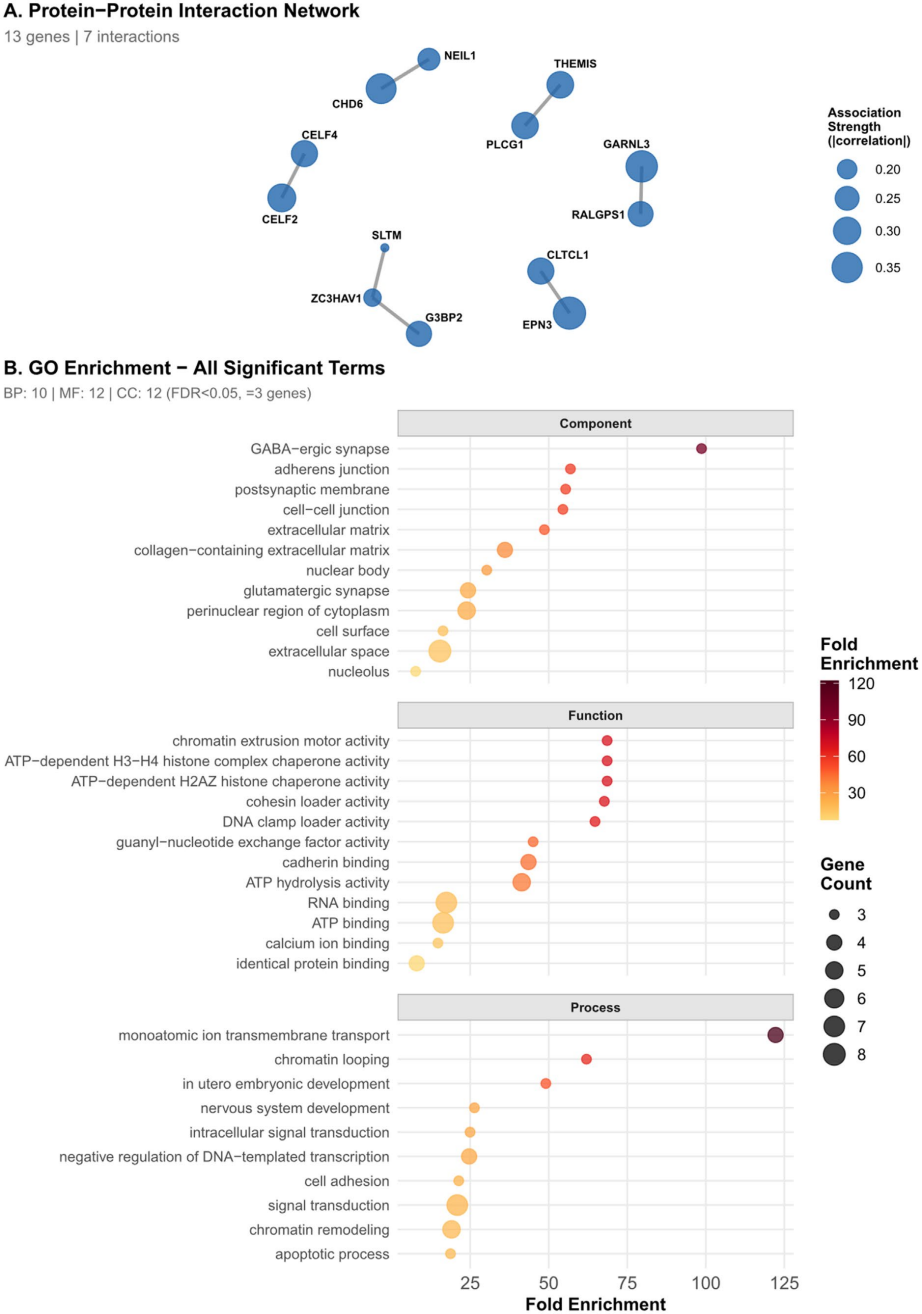
Protein–protein interaction network and functional enrichment for genes correlated with urban coverage (A) Network of 13 connected genes showing 7 protein–protein interactions (density = 0.0045). Node size represents association strength (|correlation|); edge thickness represents interaction confidence from STRING database (minimum score = 400). The remaining 43 genes showed no detectable interactions. (B) Gene Ontology enrichment analysis identified 34 significantly enriched terms (FDR < 0.05, ≥ 3 genes) across biological processes (BP: 10), molecular functions (MF: 12), and cellular components (CC: 12). Point size indicates gene count; color indicates fold enrichment.

Protein–protein interaction network analysis (Figure 3A) revealed that 13 of the 56 genes showing differentiation between urban and rural populations form an interconnected network with 7 protein–protein interactions. The network includes hub genes EPN3, GARNL3, CHD6, THEMIS, PLCG1, CLTCL1, CELF2, CELF4, G3BP2, RALGPS1, NEIL1, SLTM, and ZC3HAV1 (network density = 0.0045, average degree = 0.25). The remaining 43 genes showed no detectable protein–protein interactions in the STRING database at the medium confidence threshold (score ≥ 400).

Gene Ontology enrichment analysis (Figure 3B) identified 34 significantly enriched terms (FDR < 0.05, minimum 3 genes per term) distributed across biological processes (10 terms), molecular functions (12 terms), and cellular components (12 terms). The most significantly enriched cellular component terms were extracellular space (8 genes: CDH13, CFAP58, COL6A3, CPA6, CPN2, IL19, SPOCK1, TNN; 15.4‐fold enrichment, FDR = 6.19 × 10^−7^), perinuclear region of cytoplasm (5 genes: CDH13, EPN3, PDLIM4, SLC2A10, SYNE1; 23.9‐fold enrichment, FDR = 1.09 × 10^−5^), and GABA‐ergic synapse (3 genes: CDH13, GRID1, KIF5C; 98.6‐fold enrichment, FDR = 1.74 × 10^−5^). Molecular function enrichment revealed strong selection on ATP‐dependent processes, including ATP hydrolysis activity (5 genes: ATP13A5, ATP8B1, CHD6, KIF5C, NAV2; 41.4‐fold enrichment, FDR = 1.16 × 10^−6^), chromatin remodelling activities (3 genes: ATP13A5, CHD6, NAV2; 68.6‐fold enrichment, FDR = 3.14 × 10^−5^), and RNA binding (7 genes: C1D, CELF2, G3BP2, SLTM, SPATS2L, SYNE1, ZC3HAV1; 17.4‐fold enrichment, FDR = 1.16 × 10^−6^). Biological process enrichment highlighted monoatomic ion transmembrane transport (4 genes: ATP13A5, ATP8B1, GRID1, WWP1; 122.2‐fold enrichment, FDR = 6.19 × 10^−7^), signal transduction (7 genes: AFDN, CAMK1D, CNGB3, IL19, PLPPR3, RASA3, WWP1; 20.9‐fold enrichment, FDR = 6.19 × 10^−7^), and chromatin remodelling (5 genes: ATP13A5, CHD6, NAV2, RERE, WNK2; 19.1‐fold enrichment, FDR = 2.22 × 10^−5^). The enrichment of chromatin remodelling, ATP‐dependent processes, and synaptic functions indicates that urbanisation has imposed selection across multiple integrated biological systems rather than isolated pathways.

## 4 Discussion

Our genomic analysis reveals the signature of local adaptation in Egyptian fruit bats despite extensive gene flow between urban and rural environments. Urban and rural populations show virtually no neutral population structure, with PCA components explaining only 0.90% and 0.88% of variance, cross‐entropy analysis supporting a single genetic cluster (*K* = 1), and genome‐wide *F*_ST_ values indicating extensive gene flow. Despite this genome‐wide homogeneity, we detect strong selection signatures at 59 SNPs across 56 genes potentially underlying documented behavioural and physiological differences. This pattern, where the vast majority of markers (99.7%) are homogenised by gene flow while specific loci maintain adaptive differences, exemplifies how local adaptation can proceed in highly mobile species when selection is sufficiently strong at key genomic regions (Yeaman and Whitlock 2011; Tigano and Friesen 2016). The four outlier individuals (two urban, two rural) that appear slightly separated from the main cluster in the PCA show no pattern corresponding to geography or ecology, and when forced to *K* = 2, ancestry coefficients distribute randomly across all 185 individuals regardless of origin, further confirming the lack of neutral population structure. The observed isolation by distance pattern provides the critical spatial genetic structure necessary for adaptive divergence to be maintained.

The pattern of isolation by distance reflects a key balance that facilitates adaptation in this mobile species. Geographic distance imposes sufficient population structure on the population to avoid complete panmixia, yet allows selection to maintain adaptive differences in the face of ongoing gene flow. Such population viscosity is consistent with the Egyptian fruit bat foraging ecology: although individuals are able to navigate to foraging goals over 20 km away (Toledo et al. 2020), thereby potentially supporting extensive gene flow, roost fidelity prevents the complete genetic homogenisation that would prevent local adaptation. This balance of high mobility enabling the exchange of genes without eliminating spatial structure illustrates how adaptation may proceed in volant species despite their potential for long‐distance movements.

The persistence of adaptive variation in the face of high gene flow is consistent with recent theoretical frameworks showing that sufficiently strong selection can maintain adaptive divergence even with high levels of migration, especially where adaptation is polygenic, involving multiple loci of small effect (Yeaman and Whitlock 2011; Tigano and Friesen 2016). However, the low *p*‐values across our candidate SNPs suggest that selection coefficients are likely large enough to resist the homogenising effects of gene flow. This matches recent models showing that polygenic adaptation may proceed through subtle allele frequency shifts across many loci rather than fixation at few loci, thus enabling rapid evolution despite gene flow (Höllinger et al. 2019; Barghi et al. 2020).

The landscape genetics analysis showed that gene flow between these populations is not uniform across habitats. Urbanisation in fact connects the populations rather than fragmenting them. Of the land covers considered, cities, orchards, and forests facilitated gene flow, whereas open areas acted as a barrier to it. According to the gene flow density maps, connectivity is expected to be high within cities and among cities, moderate in rural regions, and gene flow across open habitats would be minimal. This unexpected pattern suggested that cities increased gene flow while maintaining adaptive divergence, showing that urban environments simultaneously attract bats through concentrated resources while imposing selection strong enough to overcome the homogenising effects of migration. However, tree cover also played an important role in supporting genetic connectivity, indicating that it may be the tree cover in urban areas that facilitates connectivity rather than urban areas themselves. The current landscape model builds upon the previous microsatellite‐based study by Centeno‐Cuadros et al. (2017) by incorporating the effects of artificial light intensity, continuous tree cover, and orchard proximity as landscape variables. These variables were chosen because they more directly capture the resources and conditions influencing the movement ecology of Egyptian fruit bats, such as foraging habitat and navigational cues, than the broader land‐use categories used previously. Our results show that tree cover and land cover best explain genetic connectivity, whereas artificial lighting and orchard proximity alone did not significantly predict gene flow. This refines the earlier conclusion that habitat use did not predict gene flow beyond geographic distance, and instead suggests that the specific composition of the landscape matrix matters more than urbanisation per se.

The genomic architecture of genes showing differentiation between urban and rural populations reveals modest network connectivity. Protein–protein interaction analysis identified 13 genes forming an interconnected network with 7 interactions (network density = 0.0045), while the remaining 43 genes showed no detectable interactions at medium confidence thresholds. The connected network contains genes spanning multiple functional categories, including metabolism, GTPase signalling regulators, immune and stress response genes, and RNA‐binding proteins. These hub genes show among the strongest selection signatures in the dataset, suggesting that genes under strong selection may be more likely to have characterised protein interactions, or that selection has particularly targeted genes with central roles in biological networks.

The predominance of intronic variants, 51 of 59 SNPs located within introns, lends important insights into mechanisms of rapid urban adaptation. Intronic variants can affect gene expression through several non‐mutually exclusive mechanisms. They might disrupt or create splicing regulatory elements that control splice site recognition (Baralle and Baralle 2018). Intronic variants can alter RNA secondary structures that affect splicing efficiency, create cryptic splice sites leading to alternative isoforms, or modify intronic regulatory elements such as enhancers which loop to promoters to regulate transcription (Shaul 2017; Rose 2019). Moreover, introns harbor microRNA binding sites, and variants here can affect post‐transcriptional regulation via altered miRNA‐mRNA interactions (Steri et al. 2018). This concentration of variants in introns rather than exons suggests that urban adaptation proceeds primarily through quantitative changes in gene expression rather than qualitative changes in protein function, thus allowing fine‐tuning of phenotypes without risking protein misfolding or loss of function.

We find remarkably strong selection signatures against this backdrop of regulatory evolution. CFAP58 represents the strongest signal in the entire dataset, encoding cilia and flagella associated protein, which is potentially crucial for mechanosensory perception and cellular motility. This extreme signal, in conjunction with other top candidates, namely EPN3, CACNA1G, B3GAT2, and GARNL3, indicates selection strong enough to maintain differentiation despite gene flow. These selection coefficients are of the magnitude comparable to those documented in classic examples of rapid evolution, including industrial melanism in peppered moths (Cook and Saccheri 2013) and beak size evolution in Galápagos finches (Grant and Grant 2006).

Amongst the top candidate genes, those related to neurotransmitter and sensory processing genes are overrepresented (Table 4). GRID1 has functions beyond glutamate receptor activity. It regulates burst firing of dopaminergic neurons and thus influences reward processing (Benamer et al. 2018). GRID1 also regulates forebrain circuits regulating exploratory behaviour (Andrews and Dravid 2021), thus linking neural function to the documented behavioural differences between populations. Another candidate gene is CNGB3, which codes for the beta subunit of cyclic nucleotide‐gated channels of cone photoreceptors. These channels are essential for phototransduction (Amaral et al. 2023; Gerhardt et al. 2023). Therefore, this gene is a candidate for adaptation to the light conditions of urbanisation. The concentration of selection signatures from neurotransmitter and sensory processing genes points to the significant impact of urbanisation on these systems.

This pattern of sensory system evolution shows remarkable convergence across urban taxa globally. Urban blackbirds in Europe show parallel changes in SERT serotonin transporter genes across multiple cities, with behavioural shifts toward reduced anxiety‐like behaviours (Mueller et al. 2013). Great tits across European cities independently evolved changes in DRD4 dopamine receptor variants associated with increased exploratory behaviour (Riyahi et al. 2015). White‐footed mice in New York City parks show parallel selection signatures at metabolic and immune genes across isolated populations, demonstrating convergent adaptation despite no gene flow between parks (Harpak et al. 2021). Urban Darwin's finches in the Galápagos show changes in genes affecting beak morphology and behaviour, adapting to human food sources (De León et al. 2019). Even urban‐dwelling coyotes show genomic signatures of selection on genes related to diet and immunity, paralleling changes in other urban carnivores (Kreling et al. 2025). The repeated targeting of neurotransmitter pathways (SERT, DRD4, GRID1), immune genes, and metabolic pathways across birds, mammals, and now bats reveals predictable genomic responses to urbanisation that transcend phylogenetic boundaries.

Of the metabolic genes, the second strongest signal of selection in the entire dataset was detected in the EPN3 gene, which might play a role in the dietary flexibility seen among urban‐foraging bats, as the latter have access to a significantly more varied diet compared to their rural‐foraging counterparts (Egert‐Berg et al. 2021). This gene is involved in the regulation of clathrin‐mediated endocytosis, which is essential for nutrient uptake and thus makes it a metabolic hub. Other genes involved in metabolism and showing evidence of selection in urbanised bats include ATP13A5, CAMK1D, and CHD6 (Table 4).

Gene Ontology enrichment analysis makes the functional coherence among genes under selection evident. The 56 candidate genes exhibit non‐random clustering into specific biological systems, with enrichments for some categories occurring in excess of 100‐fold above chance expectation. For example, the strong enrichment of components of GABAergic synapse (98.6‐fold) and monoatomic ion transport functions (122.2‐fold) suggests that selection has targeted neural signalling pathways in a systematic manner, rather than affecting genes randomly across the genome. Similarly, strong enrichment of ATP‐dependent chromatin remodelling activities (68.6‐fold) and RNA binding functions (17.4‐fold) reveals coordinated selection on gene regulatory machinery at multiple levels‐from chromatin structure to post‐transcriptional control. The functional clustering pattern is inconsistent with the random distribution of variants across gene functions that would be produced by neutral evolution or demographic processes but supports adaptive evolution, where urbanisation has imposed selection pressures simultaneously across interconnected biological systems (neural function, metabolic regulation, and stress response) that together enable the documented behavioural and physiological differences between populations.

The signature of selection in developmental genes might play a role in the early behavioural differences observed between urban and rural pups. The selection signature on the STRA8 gene, a retinoic acid‐responsive transcriptional regulator controlling gametogenesis (Kojima et al. 2019; Zhao et al. 2024), is significant in this context given that urban Egyptian fruit bats actually give birth approximately 2.5 weeks earlier in spring than rural bats, with this advanced parturition enabling some females to complete a second reproductive cycle in late summer (Weinberg et al. 2025). Retinoic acid signalling pathways are regulated by circadian rhythms and photoperiod (Ashton et al. 2018), and the different photoperiodic conditions between urban and rural habitats may result in different selective pressures on retinoic acid‐responsive genes like STRA8. NAV2 is a neuron navigator gene controlling neuronal migration and axon guidance and is linked to cerebellar hypoplasia, motor dysfunction, and behavioural changes in mice with loss‐of‐function mutations (Accogli et al. 2023). The selection signatures on these developmental genes suggest a genetic basis for behavioural differences in urban and rural pups, consistent with the partial retention of population‐specific behavioural traits in combination with strong maternal effects in cross‐fostered pups (Harten et al. 2021).

In addition to developmental genes, selection signatures were also observed in structural, stress response, and immune genes. Selection signatures on NEIL1, a DNA glycosylase necessary to repair oxidised bases and sustain neuronal function (Canugovi et al. 2012), and CHD6, a chromatin remodeller that increases the expression of antioxidant genes in response to oxidative stress (Moore et al. 2019), imply that oxidative stress may be a selective force in urban environments. Immune genes showing selection signatures included THEMIS, which plays a role in T‐cell development (Yang et al. 2022) and WWP1, a negative regulator of innate immune responses (Lin et al. 2013). Other structural and immune genes showing evidence of selection are listed in Table 4.

However, there are important limitations to our study. While cost‐effective for population genomics, the ddRAD‐seq approach captures only a small fraction of the genome through reduced representation sequencing (Andrews et al. 2016). ddRAD‐seq protocols can generate substantial read overlaps and adapter contamination due to imperfect size selection. This is because sequencing efficiency varies considerably depending on enzyme choice and experimental design (Lajmi et al. 2025). This reduced representation misses potentially adaptive genetic variants in unsequenced regions (Lowry et al. 2017). We lack functional validation of the identified variants; while strong selection signatures suggest functionality, we cannot confirm causality without experimental approaches, such as gene expression studies or functional assays in model systems. Reciprocal translocation experiments between urban and rural sites would quantify the fitness costs of genotype‐environment mismatch, revealing the adaptive value of allelic variants at these loci. Our cross‐sectional sampling provides only a snapshot of current genetic variation and cannot differentiate ongoing from contemporary selection or confirm the direction of evolutionary change. Temporal sampling across generations would distinguish ongoing directional selection from stable divergence, maintained through spatially varying selection (Barrett and Hoekstra 2011), and determine whether the observed patterns represent contemporary evolution or historical differentiation. Sample sizes of ~19 individuals per colony and over 90 per area, though more than sufficient to find significant effects in many population genetics analyses using thousands of SNPs (Nazareno et al. 2017; Aguirre‐Liguori et al. 2020) may have limited power to detect weak selection or rare variants, which require substantially larger samples (Ma et al. 2015; Politi et al. 2023). Also, we cannot exclude that some of the identified variants are linked to causal variants instead of being causal on their own. Indeed, fine‐mapping studies consistently show that most associated variants are in linkage disequilibrium with true causal variants rather than being the latter ones themselves (Li and Zhou 2025).

## 5 Conclusions

Our work documents that Egyptian fruit bats exhibit genetic signatures of adaptation to urbanisation despite widespread gene flow between urban and rural populations. We identified candidate SNPs across 56 genes, with selection evident in genes involved in neurotransmission, metabolism, and gene regulation. The predominance of intronic variants indicates that adaptation proceeds through regulatory changes in gene expression rather than protein‐coding changes. Network and Gene Ontology enrichment analyses revealed coordinated selection across multiple biological systems, with significant enrichment of chromatin remodelling, ATP‐dependent processes, and synaptic functions. Genetic patterns provide a foundation to understand the documented behavioural and physiological differences among urban and rural populations. For conservation management, this indicates that both urban and rural habitats provide distinct selective environments that contribute towards maintaining genetic diversity in this species. Although our reduced‐representation approach does not permit whole‐genome coverage, the candidate genes identified herein provide targets for functional validation in subsequent studies. As urbanisation continues to expand across the globe, understanding the genetic mechanisms underlying rapid adaptation to anthropogenic environments will be increasingly important for the prediction of how wildlife will continue to respond to ongoing urban growth.

## Funding

This work was supported by the European Research Council (Grant ERC‐GPS‐Bat‐679186).

## Conflicts of Interest

The authors declare no conflicts of interest.

## Supporting information


**Figure S1:** Spatial distribution map of urban and rural Egyptian fruit bat colonies sampled in this study.

**Figure S2:** Redundancy analysis (RDA) comparing light intensity and urban coverage to the SNP dataset.

**Table S1:** Levels of inbreeding and heterozygosity per individual.

**Table S2:** List of variables included in the landscape genetics analysis, their sources, assigned resistance costs, and explanation for cost allocation.

**Table S3:** Results of the Multiple Regression on Distance Matrices analysis to select best resistance costs for the land cover hypothesis.

**Table S4:** RDA results of all output SNPs located within known genes.

## Data Availability

The data for this study have been deposited in the European Nucleotide Archive (ENA) at EMBL‐EBI under accession number PRJEB104892 (https://www.ebi.ac.uk/ena/browser/view/PRJEB104892). The codes and SNPs data used in the study are available at https://figshare.com/projects/Urbanisation_drives_microevolution_in_the_Egyptian_fruit_bat_i_Rousettus_aegyptiacus_i_/273409.
