# Supplementary figures and images for: Urbanisation Drives Microevolution in the Egyptian Fruit Bat (Rousettus aegyptiacus)
Source: Evol Appl. 2026 Apr 24;19(4):e70243. doi: 10.1111/eva.70243 (PMC13108426; doi:10.1111/eva.70243)

A.

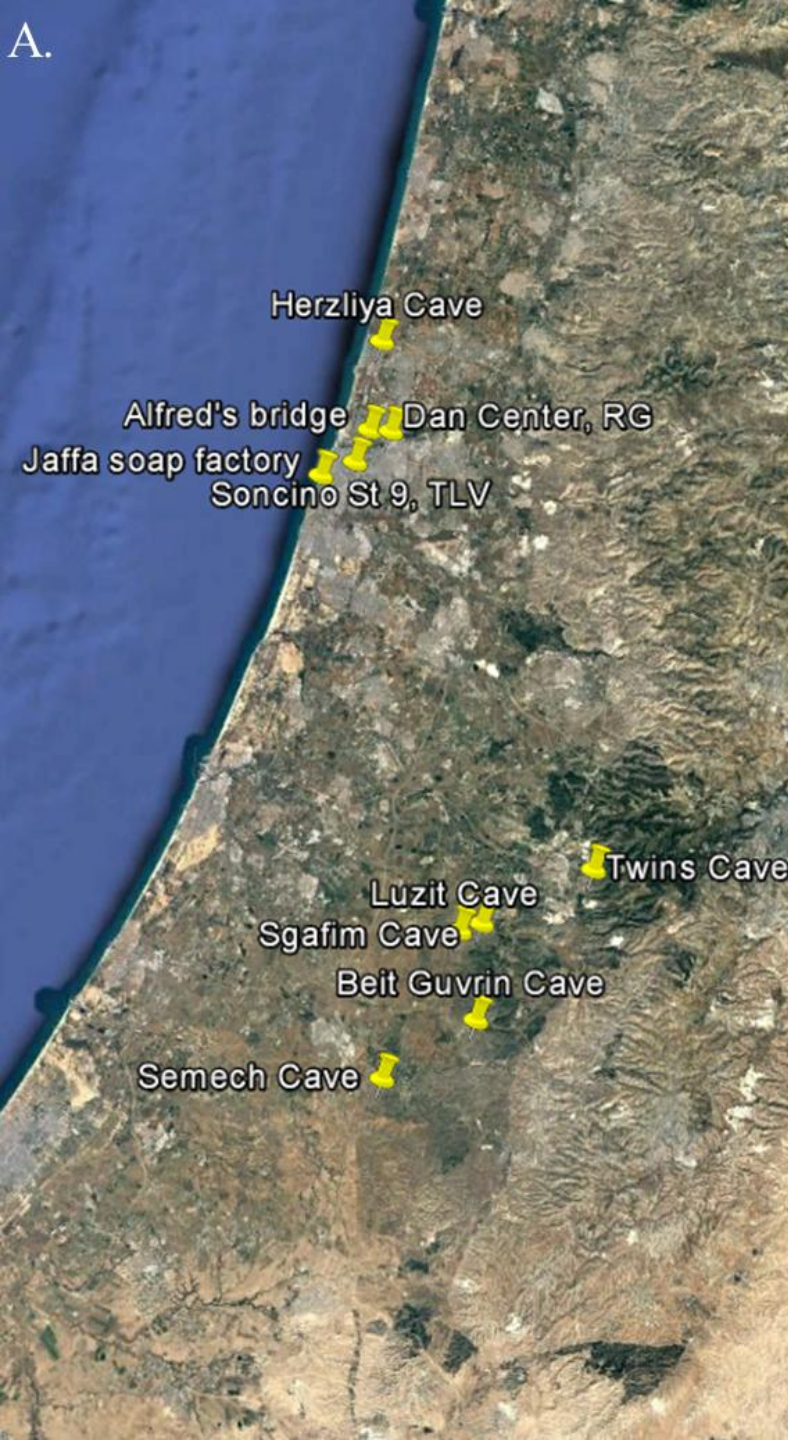

B.

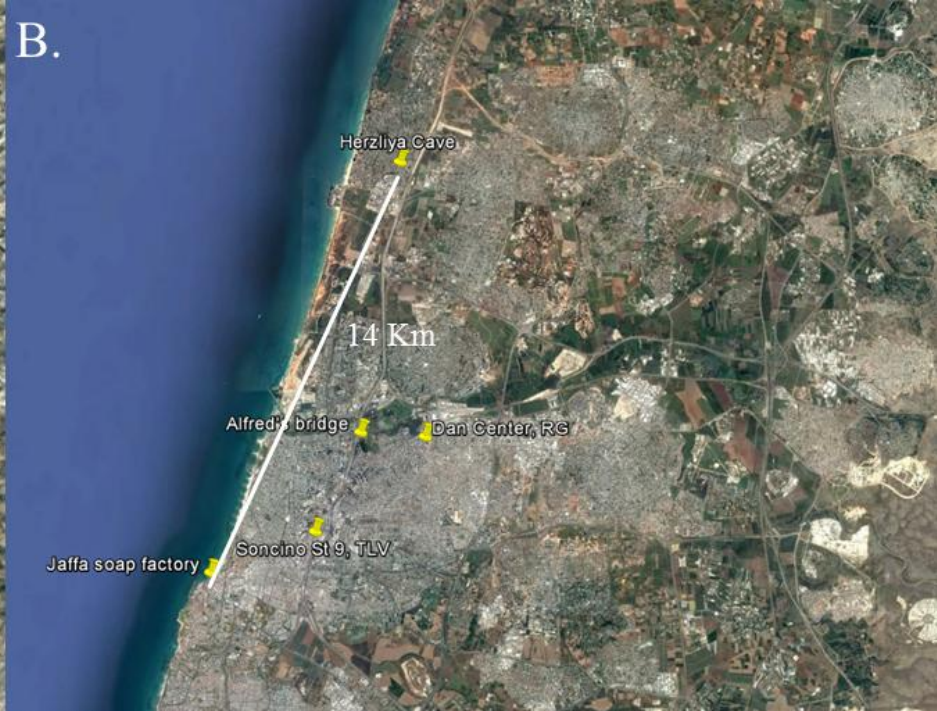

C.

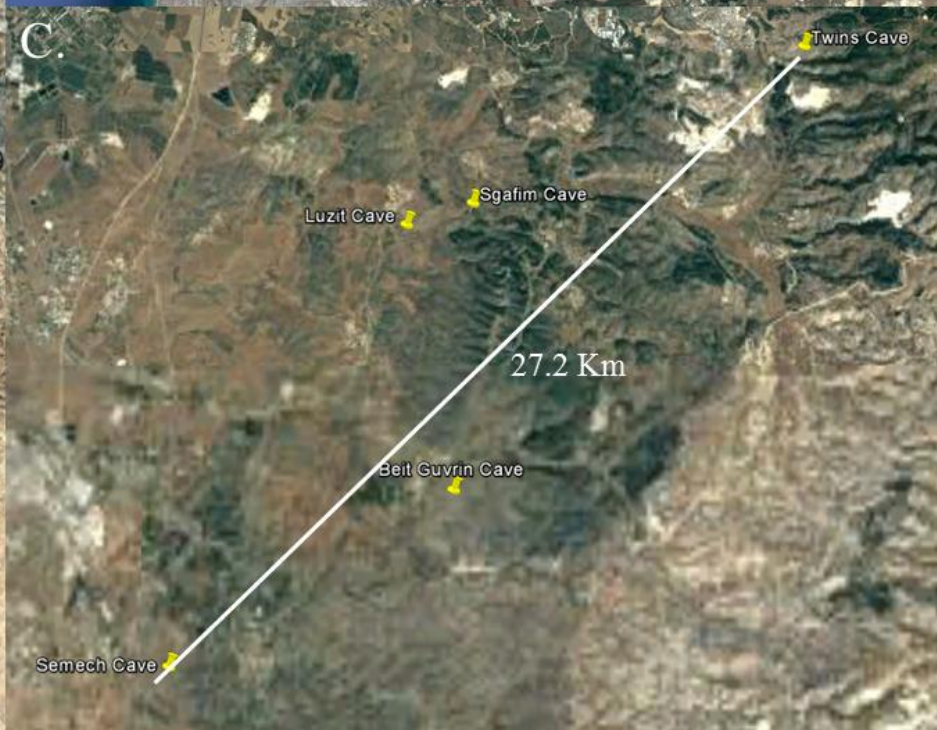

Supplement: Supplementary file 1 — Figure S1: Spatial distribution map of urban and rural Egyptian fruit bat colonies sampled in this study. [file EVA-19-e70243-s003.pdf]

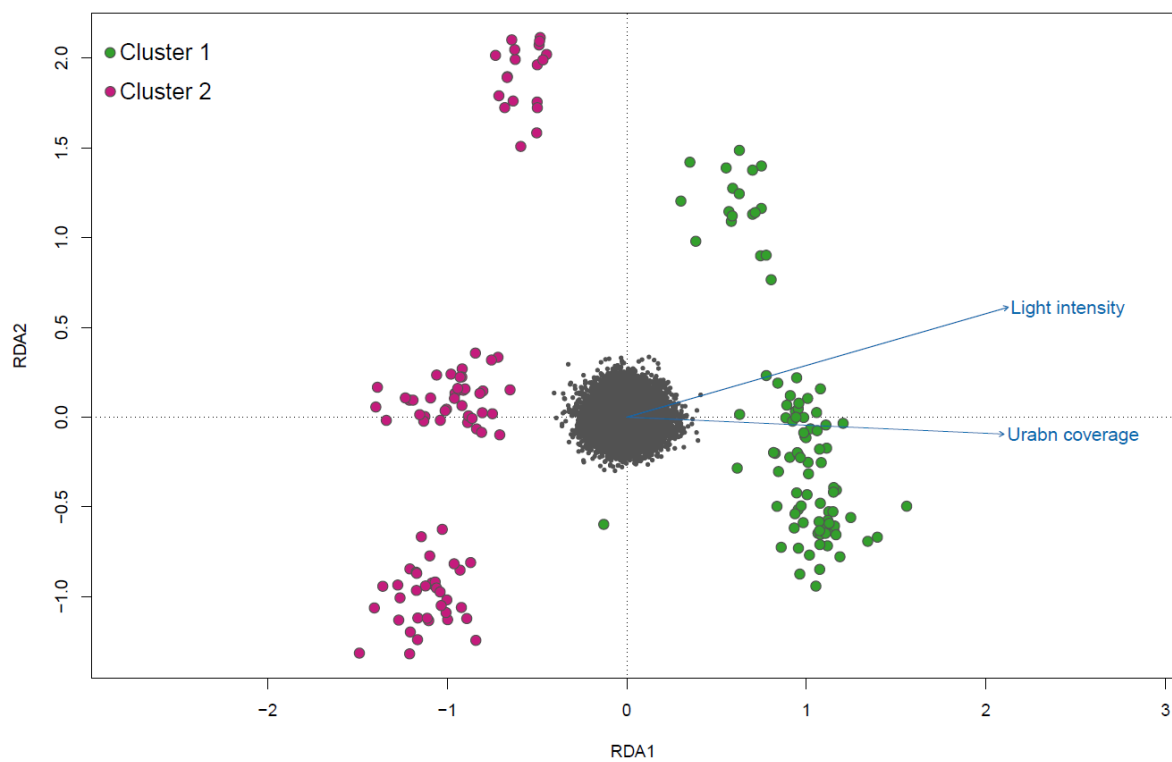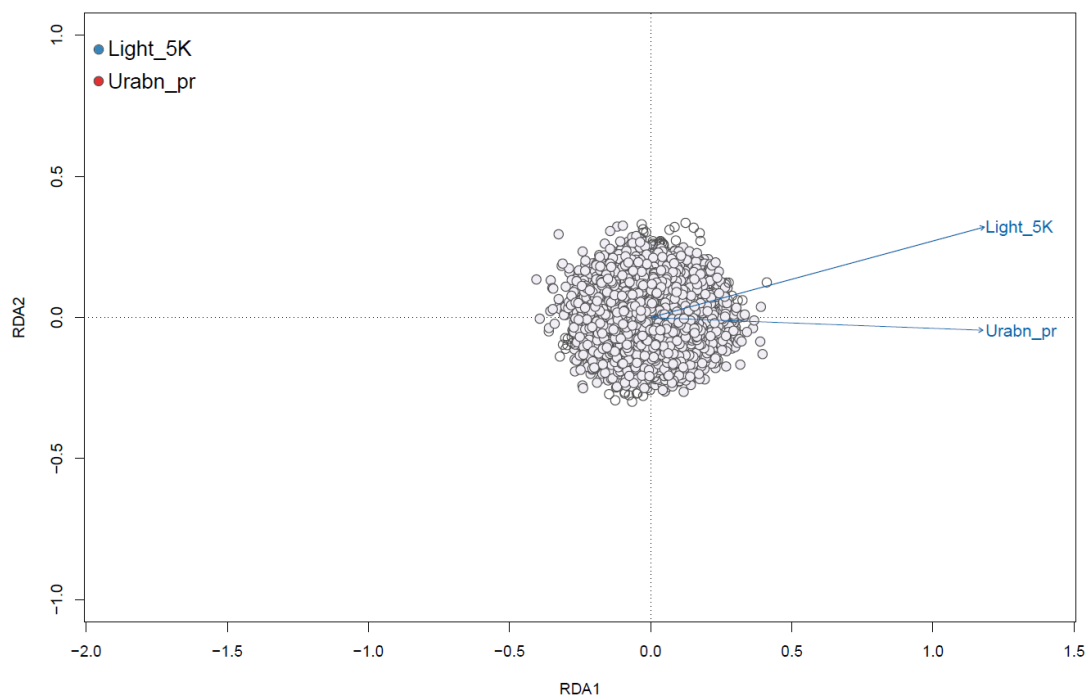

Supplement: Supplementary file 2 — Figure S2: Redundancy analysis (RDA) comparing light intensity and urban coverage to the SNP dataset. [file EVA-19-e70243-s002.pdf]
