# Supplementary material for: Urbanisation Drives Microevolution in the Egyptian Fruit Bat (Rousettus aegyptiacus)
Source: Evol Appl. 2026 Apr 24;19(4):e70243. doi: 10.1111/eva.70243 (PMC13108426; doi:10.1111/eva.70243)
Supplement: Supplementary file 3 — Table S1: Levels of inbreeding and heterozygosity per individual. [file EVA-19-e70243-s006.docx]

**Table S1**: Levels of inbreeding and heterozygosity per individual.

| **Sample ID** | **Region** | **Inbreeding** | **Heterozygosity** |
| --- | --- | --- | --- |
| A1 | Rural | 0.0108406 | 0.02279 |
| A10 | Rural | -0.0468683 | -0.06974 |
| A11 | Rural | 0.0170032 | 0.02095 |
| A12 | Rural | -0.022934 | -0.004593 |
| A13 | Rural | -0.00608154 | -0.01798 |
| A14 | Rural | -0.0131531 | -0.006906 |
| A15 | Rural | -0.00382633 | -0.02047 |
| A16 | Rural | 0.00323375 | 7.83E-05 |
| A17 | Rural | -0.000589513 | -0.01199 |
| A18 | Rural | 0.00871892 | -0.01197 |
| A19 | Rural | 0.0171588 | 0.01778 |
| A2 | Rural | -0.0039972 | 0.02156 |
| A20 | Rural | 0.00896279 | -0.006261 |
| A3 | Rural | -0.0299248 | -0.04523 |
| A4 | Rural | 0.0106281 | 0.008827 |
| A5 | Rural | -0.000556752 | 0.0155 |
| A6 | Rural | -0.0154435 | -0.01468 |
| A7 | Rural | -0.102689 | -0.1588 |
| A8 | Rural | 0.00275432 | -0.01492 |
| B1 | Rural | -0.00817221 | -0.003718 |
| B10 | Rural | -0.00757846 | -0.03209 |
| B11 | Rural | -0.0273639 | -0.02785 |
| B12 | Rural | 0.0113038 | 0.02579 |
| B13 | Rural | -0.0298128 | -0.0306 |
| B14 | Rural | -0.0241653 | -0.03621 |
| B15 | Rural | 0.0053943 | 0.005932 |
| B16 | Rural | -0.0188048 | -0.02044 |
| B17 | Rural | 0.0104509 | 0.02709 |
| B18 | Rural | -0.00601304 | -0.01882 |
| B19 | Rural | -0.00610516 | -0.006587 |
| B20 | Rural | 0.00304761 | -0.006443 |
| B3 | Rural | -0.0173018 | -0.007642 |
| B4 | Rural | 0.0027105 | 0.01818 |
| B5 | Rural | 0.00336786 | 0.01595 |
| B6 | Rural | -0.0387309 | -0.03981 |
| B7 | Rural | -0.0233347 | -0.04591 |
| B9 | Rural | 0.000525534 | 0.01975 |
| L1 | Rural | -0.00305278 | -0.004398 |
| L12 | Rural | -0.00440613 | -0.02478 |
| L13 | Rural | 0.00842928 | -0.01299 |
| L14 | Rural | -0.0110751 | -0.02623 |
| L15 | Rural | -0.00416791 | -0.01112 |
| L16 | Rural | -0.00265586 | -0.0006782 |
| L17 | Rural | -0.00675377 | -0.0216 |
| L18 | Rural | -0.0205722 | -0.01552 |
| L2 | Rural | 0.00166915 | -0.003444 |
| L20 | Rural | -0.00091037 | 0.0006758 |
| L3 | Rural | -0.0140074 | -0.0273 |
| L4 | Rural | 0.0095707 | 0.01842 |
| L5 | Rural | -0.0112901 | -0.01371 |
| L6 | Rural | -0.0110227 | -0.0086 |
| L7 | Rural | 0.0250832 | 0.01622 |
| L8 | Rural | -0.0140103 | -0.01961 |
| L9 | Rural | 0.00458602 | 0.0181 |
| S10 | Rural | -0.0100374 | -0.03037 |
| S11 | Rural | 0.00916533 | -0.006042 |
| S12 | Rural | 0.0391215 | 0.04206 |
| S13 | Rural | -0.0160355 | -0.03457 |
| S14 | Rural | -0.0193768 | -0.03462 |
| S15 | Rural | -0.00776622 | 0.003866 |
| S16 | Rural | 0.0108374 | 0.008222 |
| S17 | Rural | -4.76E-06 | -0.01491 |
| S18 | Rural | -0.000364916 | 0.003322 |
| S19 | Rural | -0.000312962 | 0.008524 |
| S2 | Rural | 0.00392456 | 0.005171 |
| S20 | Rural | -0.00183441 | 0.01265 |
| S3 | Rural | -0.00400194 | -0.007657 |
| S4 | Rural | 0.00129814 | -0.001661 |
| S5 | Rural | -0.0967187 | -0.1551 |
| S6 | Rural | -0.0102341 | -0.01075 |
| S7 | Rural | -0.0174037 | -0.02012 |
| S8 | Rural | 0.0128927 | 0.01588 |
| S9 | Rural | 0.0619255 | 0.06478 |
| T1 | Rural | -0.0121754 | -0.03479 |
| T10 | Rural | 0.0117864 | 0.01722 |
| T11 | Rural | -0.000944374 | -0.0111 |
| T12 | Rural | -0.00155752 | -0.002371 |
| T13 | Rural | -0.0187255 | -0.02053 |
| T14 | Rural | 0.00745954 | 0.0101 |
| T15 | Rural | 0.00217644 | 0.006715 |
| T16 | Rural | -0.0164988 | -0.009814 |
| T17 | Rural | -0.00597704 | 0.0008456 |
| T18 | Rural | -0.016335 | -0.03542 |
| T19 | Rural | 0.00471843 | -0.001011 |
| T2 | Rural | 0.00686133 | -0.008267 |
| T20 | Rural | -0.00972308 | -0.03614 |
| T3 | Rural | -0.00406895 | 0.01437 |
| T4 | Rural | 0.000731448 | -0.02025 |
| T5 | Rural | 0.0114117 | 0.02195 |
| T6 | Rural | -0.00818122 | -0.004894 |
| T7 | Rural | 0.00260849 | 0.003744 |
| T9 | Rural | -0.00607034 | -0.008356 |
| 1 | Urban | -0.018578 | -0.04542 |
| 10 | Urban | 0.00680634 | 0.02644 |
| 11 | Urban | -0.0109542 | -0.02986 |
| 12 | Urban | -0.0421657 | -0.05669 |
| 13 | Urban | -0.0471901 | -0.05889 |
| 14 | Urban | -0.0122494 | -0.008992 |
| 15 | Urban | -0.00977243 | -0.01117 |
| 16 | Urban | -0.00597771 | -0.003654 |
| 17 | Urban | -0.00699688 | -0.01277 |
| 18 | Urban | -0.0195326 | -0.0266 |
| 19 | Urban | -0.0191972 | -0.03563 |
| 20 | Urban | -0.00863035 | -0.02055 |
| 3 | Urban | -0.00510211 | 0.0007397 |
| 4 | Urban | -0.00390416 | 0.003548 |
| 5 | Urban | 0.00306755 | 0.005929 |
| 6 | Urban | 0.00845351 | 0.007455 |
| 8 | Urban | -0.0333994 | -0.06176 |
| 9 | Urban | -0.0110559 | -0.01335 |
| G10 | Urban | 0.00365017 | 0.003577 |
| G11 | Urban | -0.00201056 | 0.001549 |
| G12 | Urban | 0.00163804 | 0.01643 |
| G13 | Urban | -0.0126386 | -0.02647 |
| G14 | Urban | -0.0890824 | -0.1237 |
| G15 | Urban | -0.00746879 | -0.004149 |
| G16 | Urban | -0.00427636 | 0.01945 |
| G17 | Urban | -0.0424614 | -0.06276 |
| G18 | Urban | -0.0784164 | -0.1304 |
| G2 | Urban | -0.0294871 | -0.0338 |
| G20 | Urban | -0.00488039 | -0.01091 |
| G3 | Urban | -0.0421506 | -0.05625 |
| G4 | Urban | 0.0106597 | 0.005062 |
| G5 | Urban | -0.00166094 | 0.008465 |
| G6 | Urban | 0.0390954 | 0.01693 |
| G7 | Urban | -0.0127483 | -0.01545 |
| G8 | Urban | -0.0160369 | -0.02946 |
| G9 | Urban | -0.0396623 | -0.02403 |
| H1 | Urban | 0.00922352 | 0.009529 |
| H10 | Urban | -0.0277929 | -0.03537 |
| H11 | Urban | 0.00394473 | -0.001921 |
| H12 | Urban | 0.00241769 | 0.008231 |
| H13 | Urban | 0.00988584 | 0.005007 |
| H14 | Urban | 0.010161 | -0.006479 |
| H15 | Urban | -0.00441758 | 0.001491 |
| H16 | Urban | 0.0408622 | 0.03019 |
| H17 | Urban | -0.00153196 | -0.01989 |
| H18 | Urban | 0.0143021 | 0.01216 |
| H19 | Urban | -0.00377782 | -0.0208 |
| H2 | Urban | -0.0145256 | -0.002225 |
| H20 | Urban | 0.0211598 | 0.03298 |
| H3 | Urban | -0.00930947 | -0.003814 |
| H4 | Urban | 0.00926796 | 0.001706 |
| H5 | Urban | -0.0171726 | -0.003536 |
| H6 | Urban | 0.0156343 | 0.03198 |
| H8 | Urban | -0.0580586 | -0.06616 |
| H9 | Urban | -0.0174336 | -0.003267 |
| J1 | Urban | 0.027848 | 0.01647 |
| J10 | Urban | -0.00478927 | -0.004126 |
| J11 | Urban | -0.00464619 | -0.001289 |
| J12 | Urban | -0.0163486 | 0.00347 |
| J13 | Urban | 0.0650558 | 0.05274 |
| J14 | Urban | 0.0138481 | -0.004172 |
| J15 | Urban | -0.0253758 | -0.003084 |
| J16 | Urban | -0.000406801 | 0.003514 |
| J17 | Urban | -0.0225351 | -0.03339 |
| J18 | Urban | -0.00430027 | 0.001151 |
| J19 | Urban | 0.0258274 | 0.0174 |
| J2 | Urban | 0.0027942 | 0.002155 |
| J20 | Urban | -0.00138447 | 0.01796 |
| J3 | Urban | -0.00619315 | -0.003459 |
| J4 | Urban | -0.00595535 | -0.01871 |
| J5 | Urban | -0.00648187 | -0.01076 |
| J6 | Urban | -0.00804214 | -0.02383 |
| J7 | Urban | 0.0072327 | 0.006608 |
| J9 | Urban | 0.00210226 | 0.01587 |
| R1 | Urban | -0.00574042 | -0.02986 |
| R10 | Urban | -0.00922993 | -0.009089 |
| R11 | Urban | 0.0229413 | 0.02667 |
| R12 | Urban | -0.0191466 | -0.02005 |
| R14 | Urban | -0.0041645 | -0.02146 |
| R15 | Urban | -0.01205 | -0.02065 |
| R16 | Urban | 0.000308599 | 0.002071 |
| R17 | Urban | -0.0209114 | -0.02573 |
| R18 | Urban | -0.00252083 | -0.01473 |
| R19 | Urban | 0.00551933 | 0.03063 |
| R2 | Urban | -0.0248942 | -0.03069 |
| R20 | Urban | 0.00552445 | 0.002465 |
| R3 | Urban | -0.00912509 | -0.01796 |
| R4 | Urban | -0.0107594 | -0.02902 |
| R5 | Urban | -0.0976587 | -0.163 |
| R6 | Urban | -0.0269356 | -0.03794 |
| R7 | Urban | -0.017222 | -0.04295 |
| R8 | Urban | -0.207644 | -0.3409 |
| R9 | Urban | -0.0119389 | -0.02393 |
