# Supplementary material for: Urbanisation Drives Microevolution in the Egyptian Fruit Bat (Rousettus aegyptiacus)
Source: Evol Appl. 2026 Apr 24;19(4):e70243. doi: 10.1111/eva.70243 (PMC13108426; doi:10.1111/eva.70243)
Supplement: Supplementary file 4 — Table S2: List of variables included in the landscape genetics analysis, their sources, assigned resistance costs, and explanation for cost allocation. [file EVA-19-e70243-s001.docx]

**Table S2**: List of variables included in the landscape genetics analysis, their sources, assigned resistance costs, and explanation for cost allocation.

| **Variable** | **Source map** | **Cell size** | **Costs** | **Explanation** |
| --- | --- | --- | --- | --- |
| Artificial light | BlackMarble 2016 NASA (<https://blackmarble.gsfc.nasa.gov>) | 458 m | 1-100 | costs increase with light levels - light avoidance |
| Distance to orchards | Israel land cover map 2018 DMT18 (<https://www.hamaarag.org.il/>) | 25 m | 1-101 | costs increase with distance to orchards - forages on fruits from orchards |
| Tree cover | Tree canopy cover for the year 2000 from Hansen Global Forest Change (https://earthenginepartners.appspot.com/science-2013-global-forest/download_v1.2.html) | 25 m | 1-100 | costs decrease with tree cover - use trees for navigation, roosting and foraging |
| Land cover 1 | Israel land cover map 2018 DMT18 | 25 m | 1-orchards; 10-native forest; 20-water; 30-conifer; 50-urban; 70-shrub; 80-grassland & arable; 100-bare | preferentially associated with orchards and trees, not likely to cross bare areas or use arable land or grasslands, rural bats may avoid urban areas |
| Land cover 2 | Israel land cover map 2018 DMT18 | 25 m | 1-orchards; 10-native forest; 20-water; 30-conifer & urban; 70-shrub; 80-grassland & arable; 100-bare | preferentially associated with orchards and trees, not likely to cross bare areas or use arable land or grasslands, rural bats may prefer non-urban areas but do not avoid them |
| Land cover 3 | Israel land cover map 2018 DMT18 | 25 m | 1-orchards; 10-native forest & urban; 20-water; 30-conifer; 70-shrub; 80-grassland & arable; 100-bare | preferentially associated with orchards and trees, not likely to cross bare areas or use arable land or grasslands, urban areas no barrier to movement |
