# Supplementary material for: Urbanisation Drives Microevolution in the Egyptian Fruit Bat (Rousettus aegyptiacus)
Source: Evol Appl. 2026 Apr 24;19(4):e70243. doi: 10.1111/eva.70243 (PMC13108426; doi:10.1111/eva.70243)
Supplement: Supplementary file 5 — Table S3: Results of the Multiple Regression on Distance Matrices analysis to select best resistance costs for the land cover hypothesis. [file EVA-19-e70243-s004.docx]

**Table S3**: Results of the Multiple Regression on Distance Matrices analysis to select best resistance costs for the land cover hypothesis, with the selected variable highlighted in bold.

| **Variables** | **F** | **R^2^** | **P** |
| --- | --- | --- | --- |
| F_ST_ ~ land cover 1 | 2.3 | 0.05 | 0.115 |
| F_ST_ ~ land cover 2 | 6.5 | 0.132 | 0.016 |
| **F_ST_ ~ land cover 3** | **9.2** | **0.177** | **0.015** |
