# Supplementary material for: Urbanisation Drives Microevolution in the Egyptian Fruit Bat (Rousettus aegyptiacus)
Source: Evol Appl. 2026 Apr 24;19(4):e70243. doi: 10.1111/eva.70243 (PMC13108426; doi:10.1111/eva.70243)
Supplement: Supplementary file 6 — Table S4: RDA results of all output SNPs located within known genes. [file EVA-19-e70243-s005.docx]

**Table S4**: RDA results of all output SNPs located within known genes.

| locus | Urabn_pr | Urabn_pr | Chromo | Pos | Gene | WhereOnGene |
| --- | --- | --- | --- | --- | --- | --- |
| 3521:22:+_C | -0.283908604 | 8.99200928765707e-05 | NW_023416284.1 | 1645844 | ARHGEF16 | Exon |
| 8667:82:+_T | 0.147706594 | 0.044810484 | NW_023416284.1 | 4472452 | RERE | Intron |
| 18241:173:-_C | -0.269956532 | 0.000202279 | NW_023416284.1 | 10195377 | ACTL8 | Intron |
| 109863:22:+_T | 0.279816061 | 0.000114573 | NW_023416284.1 | 117984835 | B3GAT2 | Intron |
| 133390:40:-_T | 0.289034717 | 6.60308669557703e-05 | NW_023416284.1 | 157760630 | THEMIS | Intron |
| 146292:110:+_G | 0.285830704 | 8.01443309623205e-05 | NW_023416284.1 | 175640338 | SYNE1 | Intron |
| 158349:210:-_A | -0.286932022 | 7.50015191852006e-05 | NW_023416284.1 | 184816348 | AFDN | Intron |
| 200157:56:+_A | 0.266818769 | 0.000241307 | NW_023416285.1 | 49857614 | G3BP2 | Intron |
| 228908:156:+_G | -0.322665381 | 7.48619716774253e-06 | NW_023416285.1 | 92578832 | CAMK1D | Intron |
| 230368:55:-_C | -0.309462266 | 1.81668833845972e-05 | NW_023416285.1 | 93688408 | CELF2 | Intron |
| 277413:129:+_G | -0.263960929 | 0.000282843 | NW_023416286.1 | 47020353 | RASA3 | Intron |
| 328695:74:-_G | 0.272547931 | 0.000174569 | NW_023416287.1 | 9953664 | SLC2A10 | Intron |
| 333604:188:+_T | -0.34329366 | 1.71979360141212e-06 | NW_023416287.1 | 13588776 | CHD6 | Intron |
| 333869:55:-_T | 0.286954089 | 7.49017073444e-05 | NW_023416287.1 | 13778034 | PLCG1 | Exon |
| 381397:10:-_A | -0.280835294 | 0.000107902 | NW_023416287.1 | 67866883 | CELF4 | Intron |
| 381466:92:+_C | 0.289729033 | 6.32970418031245e-05 | NW_023416287.1 | 67903687 | CELF4 | Intron |
| 394283:147:-_T | 0.287899559 | 7.07404309415982e-05 | NW_023416287.1 | 82544209 | ATP8B1 | Intron |
| 458930:110:+_G | 0.18199149 | 0.013163503 | NW_023416288.1 | 55921645 | ZC3HAV1 | Intron |
| 461371:50:-_T | -0.289260911 | 6.51282991446144e-05 | NW_023416288.1 | 58341661 | STRA8 | Intron |
| 509377:108:-_A | -0.278098167 | 0.000126697 | NW_023416289.1 | 26273092 | CDH13 | Intron |
| 561490:143:+_C | 0.303086015 | 2.74633844727103e-05 | NW_023416290.1 | 1126372 | CNGB3 | Intron |
| 561510:59:-_C | 0.292717904 | 5.26971934468606e-05 | NW_023416290.1 | 1135523 | CNGB3 | Intron |
| 561681:244:+_A | 0.347687185 | 1.23989710413785e-06 | NW_023416290.1 | 1228324 | WWP1 | Intron |
| 571692:17:-_C | -0.168956917 | 0.021502658 | NW_023416290.1 | 10088784 | CPA6 | Intron |
| 674571:194:+_C | 0.268110897 | 0.000224456 | NW_023416291.1 | 54641219 | PLPPR3 | Intron |
| 680651:161:-_A | -0.279576982 | 0.000116193 | NW_023416292.1 | 4244173 | IL19 | Intron |
| 695989:86:-_G | 0.26482697 | 0.000269603 | NW_023416292.1 | 18249638 | TNN | Intron |
| 767621:114:+_A | 0.158370145 | 0.0313129 | NW_023416295.1 | 1801764 | RAMP1 | Intron |
| 768254:84:-_T | 0.277263329 | 0.000133013 | NW_023416295.1 | 2144797 | COL6A3 | Intron |
| 792562:129:+_G | -0.308392827 | 1.94837725217103e-05 | NW_023416295.1 | 27547726 | SPATS2L | Intron |
| 792570:27:-_G | -0.34346853 | 1.69770429642867e-06 | NW_023416295.1 | 27555659 | SPATS2L | Intron |
| 814296:146:-_T | -0.340816224 | 2.06371958777022e-06 | NW_023416295.1 | 64419786 | KIF5C | Intron |
| 893186:59:-_G | -0.291535896 | 5.66723432171815e-05 | NW_023416295.1 | 170513115 | NPTXR | Intron |
| 893778:22:+_C | -0.343062001 | 1.74947852776395e-06 | NW_023416295.1 | 170804571 | SYNGR1 | Intron |
| 925323:186:-_C | 0.371841601 | 1.87425831835554e-07 | NW_023416306.1 | 6671496 | GARNL3 | Intron |
| 925434:89:-_C | 0.263439053 | 0.000291111 | NW_023416306.1 | 6732252 | RALGPS1 | Intron |
| 984133:52:+_T | -0.151289598 | 0.039815162 | NW_023416306.1 | 79161170 | WNK2 | Intron |
| 986756:113:+_A | -0.226070112 | 0.001973421 | NW_023416306.1 | 81547114 | NEIL1 | Intron |
| 1021196:150:-_C | 0.145751466 | 0.047750562 | NW_023416306.1 | 127710028 | SLTM | Intron |
| 1055250:93:+_T | -0.263439551 | 0.000291103 | NW_023416306.1 | 164606894 | DGLUCY | Intron |
| 1073586:81:-_G | 0.284232981 | 8.81955595477993e-05 | NW_023416307.1 | 1822842 | IGSF5 | Intron |
| 1106304:101:+_A | -0.264673104 | 0.000271912 | NW_023416307.1 | 47647764 | ATP13A5 | Intron |
| 1107182:53:-_C | 0.148758094 | 0.043292905 | NW_023416307.1 | 48455085 | CPN2 | Intron |
| 1113241:90:+_C | 0.2832858 | 9.33201022648336e-05 | NW_023416307.1 | 54881449 | GTF2E1 | Intron |
| 1152961:99:+_A | 0.308555044 | 1.92783799993678e-05 | NW_023416307.1 | 119031324 | SSUH2 | Intron |
| 1215091:104:+_C | -0.294241714 | 4.79585065259391e-05 | NW_023416308.1 | 10984121 | CCDC60 | Exon |
| 1216441:37:-_A | 0.281013434 | 0.000106774 | NW_023416308.1 | 12208813 | CLTCL1 | Exon |
| 1286476:38:+_A | -0.280634643 | 0.000109186 | NW_023416308.1 | 90557700 | NAV2 | Intron |
| 1298243:134:+_T | 0.288854974 | 6.67564380228871e-05 | NW_023416308.1 | 106876588 | CD82 | Intron |
| 1323715:12:-_C | -0.324433942 | 6.62666371237617e-06 | NW_023416309.1 | 3775783 | GRID1 | Intron |
| 1354717:119:+_A | 0.20251301 | 0.00570085 | NW_023416309.1 | 41830880 | SH3PXD2A | Intron |
| 1355458:139:+_T | -0.392004514 | 3.42561381395345e-08 | NW_023416309.1 | 42425385 | CFAP58 | Intron |
| 1413855:115:-_C | 0.383467519 | 7.13392306349024e-08 | NW_023416309.1 | 94522804 | CACNA1G | Intron |
| 1413993:171:+_T | 0.388548596 | 4.62168801794677e-08 | NW_023416309.1 | 94581635 | EPN3 | Exon |
| 1487971:6:+_T | 0.20682353 | 0.004733322 | NW_023416310.1 | 43815738 | C1D | Intron |
| 1548327:85:+_C | 0.28215451 | 9.98060296610389e-05 | NW_023416310.1 | 99464705 | SPOCK1 | Intron |
| 1553185:68:+_C | 0.309447332 | 1.81846788457564e-05 | NW_023416310.1 | 103130059 | PDLIM4 | Intron |
| 1565511:61:+_C | -0.305259119 | 2.38811028583953e-05 | NW_023416311.1 | 414768 | HS3ST4 | Intron |
| 1567842:22:+_C | -0.15611651 | 0.033833473 | NW_023416311.1 | 1645182 | CDR2 | Intron |
